# How Will Global Environmental Changes Affect the Growth of Alien Plants?

**DOI:** 10.3389/fpls.2016.01623

**Published:** 2016-11-01

**Authors:** Jujie Jia, Zhicong Dai, Feng Li, Yanjie Liu

**Affiliations:** ^1^State Key Laboratory of Urban and Regional Ecology, Research Center for Eco-environmental Sciences, Chinese Academy of SciencesBeijing, China; ^2^University of Chinese Academy of SciencesBeijing, China; ^3^Institute of Environment and Ecology and Academy of Environmental Health and Ecological Security, School of the Environment and Safety Engineering, Jiangsu UniversityZhenjiang, China; ^4^Ecology, Department of Biology, University of KonstanzKonstanz, Germany

**Keywords:** alien plants, climate change, life forms, meta-analysis, novel environmental conditions, photosynthetic pathways, functional traits

## Abstract

Global environmental changes can create novel habitats, promoting the growth of alien plants that often exhibit broad environmental tolerance and high phenotypic plasticity. However, the mechanisms underlying these growth promotory effects are unknown at present. Here, we conducted a phylogenetically controlled meta-analysis using data from 111 published studies encompassing the responses of 129 alien plants to global warming, increased precipitation, N deposition, and CO_2_ enrichment. We compared the differences in the responses of alien plants to the four global environmental change factors across six categories of functional traits between woody and non-woody life forms as well as C3 and C4 photosynthetic pathways. Our results showed that all four global change factors promote alien plant growth. Warming had a more positive effect on C4 than C3 plants. Although the effects of the four factors on the functional traits of alien plants were variable, plant growth was mainly promoted via an increase in growth rate and size. Our data suggest that potential future global environmental changes could further facilitate alien plant growth.

## Introduction

Conservative estimates suggest that at least 3.9% of global vascular flora have been successfully naturalized in newly introduced regions (van Kleunen et al., 2015). Some species have since become invasive and spread rapidly, thereby exerting a negative impact on native biodiversity and ecosystem functions and services (Vila et al., 2011; Qi et al., 2014; Gallardo et al., 2016). Plant invasion is predicted to increase with human globalization (Seebens et al., 2015; van Kleunen et al., 2015). Elevated temperatures, altered precipitation, enhanced nitrogen (N) deposition, and increased atmospheric CO_2_ concentrations are important environmental influences on ecosystems, ranging from the species level to ecosystem level (Stevens et al., 2004; Xu et al., 2014; Dieleman et al., 2015; Hautier et al., 2015; Xu et al., 2015, 2016). Additionally, plant invasion may be strongly affected by the major components of global environmental change (Bradley et al., 2010). The associations between plant invasion and global environmental change remain an emerging topic of interest in the fields of ecology and invasion biology (Bradley et al., 2010; Sheppard et al., 2014; Leishman and Gallagher, 2015; Seebens et al., 2015).

Global environmental changes could create novel environments and directly increase the availability of plant resources. Alien plants often exhibit broad environmental tolerance (Vilà et al., 2007; Hellmann et al., 2008; Scasta et al., 2015) and high phenotypic plasticity (Richards et al., 2006; Davidson et al., 2011; Si et al., 2014), facilitating their successful growth in novel environments with high resource availability (Davis et al., 2000; Pyšek and Richardson, 2007; Dostal et al., 2013). Numerous experimental studies have demonstrated that global environmental changes promote plant invasion. For instance, a recent meta-analysis comparing the growth performance response to global environmental changes (increased temperatures, increased precipitation, N deposition, and atmospheric CO_2_ concentrations) between 74 invasive alien and 117 native plants found that these changes favor the performance of invasive alien plant species over that of native plants (Liu et al., unpublished). However, the mechanisms by which these global environmental change factors promote alien plant growth are yet to be established.

Interestingly, alien plant species classified within different functional groups (e.g., life forms and photosynthetic pathways) may show different responses to global environmental changes (Scasta et al., 2015). For example, the biomass of woody plants increases more than that of herbaceous plants in response to warming (Lin et al., 2010). C4 plants are more drought-tolerant and show favorable responses to CO_2_ enrichment and warming (Lara and Andreo, 2011; Yamori et al., 2014). Moreover, many potentially important functional traits of species appear to promote the invasiveness of alien plants. To further explore this finding, a recent meta-analysis (van Kleunen et al., 2010) compared the pair-wise trait differences of 125 invasive and 196 non-invasive plant species. The study showed that invasiveness of alien plants is associated with performance-related traits (physiology, leaf area allocation, shoot allocation, growth rate, size, and fitness). Clarification of the responses to global environmental changes of functional groups (life forms and photosynthetic pathways) and different functional traits could therefore help determine how global changes facilitate alien plant growth.

Meta-analysis is an established tool that provides information to answer important ecological questions for organisms with different traits or belonging to diverse functional groups (Osenberg et al., 1997, 1999; Scasta et al., 2016). In the current study, we conducted a phylogenetically controlled meta-analysis using data from 111 published studies encompassing 129 alien species. Specifically, we compared the differences in response to global environmental changes across six categories of functional traits between woody and non-woody life forms and between C3 and C4 photosynthetic pathways of alien plants. The following research issues were addressed: (1) the extent of differences in plant functional groups (i.e., between woody and non-woody or C3 and C4 alien plants) in terms of response to increased temperature, precipitation, N deposition, and atmospheric CO_2_ concentrations and (2) extent of variations in alien plant traits based on these global changes.

## Materials and Methods

### Data Compilation

To identify studies reporting trait responses of alien plants to global changes, we searched the ISI Web of Science^1^ and Google Scholar using the keyword combination “climate change” OR “global change” OR “warm^∗^” OR “temperature” OR “nitrogen” OR “nitrogen deposition” OR “CO_2_” OR “carbon dioxide” OR “precipitation” OR “watering” OR “drought” OR “rainfall” AND “invasive” OR “alien” OR “non-native.” We additionally searched CNKI^2^ and included studies published in the Chinese language. All published records from 1980 to June 30, 2015, were examined and the results limited to those from studies on plants. Our searches retrieved 1,036 records, including both peer-reviewed literature and dissertations. Each publication was individually assessed and retained if the following three criteria were met:

(1) At least one plant species was identified as “alien” in the study location.
(2) The effects of manipulating at least one of the four different components of global environmental change (mean levels of precipitation, temperature, atmospheric CO_2_ concentration or N deposition) on alien plants were reported.
(3) Mean values, sample sizes, and variances for traits related to physiology (i.e., photosynthetic rate), light interception (i.e., leaf area), shoot allocation (i.e., inverse of root allocation), growth rate, plant size, and fitness (i.e., survival and reproduction) of each species were documented.

In total, 111 published studies covering 129 alien plant species met the criteria (see Supplementary Materials and Methods S1 for all publications).

We recorded the means, measures of variability and sample sizes of the above traits. All data were extracted directly from the text, tables or figures using software Image J 1.47v (Rasband, 2013). The following criteria were applied to extract data for each study:

(1) We considered the ambient level of an environmental change factor (i.e., precipitation, temperature, atmospheric CO_2_ concentration, and soil N) as the “control” and elevated level of the same factor as the “treatment” group. If the ambient level was not clearly identified in a study, the lower level was taken as the “control.” In the cases of precipitation, some studies imposed drought treatment. In such cases, we considered the normal water level as “treatment” to facilitate comparisons with the other global change factors. Since the global change for precipitation is likely to increase in some regions and decrease in others, we additionally performed a separate analysis for studies involving increased and decreased precipitation (Supplementary Figure S1; Discussion).
(2) In cases where trait measures were reported for different time-points from the same experiment, we selected the longest duration of study.
(3) Since only 24 of 111 total publications in our meta-analysis manipulated more than one global environment change factor, we did not account for interactions between these components. Thus, when more than one factor was manipulated in an experiment, we used the performance measures corresponding to a single focal component, considering the ambient levels of other components.
(4) When competition was manipulated in an experiment, we included data on target plants growing under both competitive and non-competitive conditions.

### Effect Size and Variance Computation

For response variables of individual traits per species in each study, we calculated the log response ratio (ln *R*) as the effect size to determine the effects of global environmental changes on different traits of alien plants. The log response ratio was calculated using the following equation (1) (Hedges et al., 1999):

(1) $$\ln R=\text{Ln}\left(\frac{\overset{-}{{\text{X}}_{\text{t}}}}{\overset{-}{{\text{X}}_{\text{c}}}}\right)=\ln(\overset{-}{{\text{X}}_{\text{t}}})-\ln(\overset{-}{{\text{X}}_{\text{c}}})$$

where 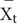 and 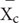 are the mean values of each trait measured in the treatment (t) and control (c) groups, respectively. Variance of ln *R* was calculated using the following equation (2) (Hedges et al., 1999):

(2) $$v\ln\text{R}=\frac{{\left({\text{SD}}_{\text{c}}\right)}^{2}}{{\text{N}}_{\text{c}}{\left(\overset{-}{{\text{X}}_{\text{c}}}\right)}^{2}}+\frac{{\left({\text{SD}}_{\text{t}}\right)}^{2}}{N_{\text{t}}{\left(\overset{-}{{\text{X}}_{\text{t}}}\right)}^{2}}$$

where N_t_, N_c_, SD_t_, SD_c_, 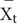, and 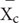 represent sample sizes, standard deviations and mean values for traits measured in the treatment and control groups, respectively. A negative value of ln *R* indicates a decrease in plant trait response to an increase in an environmental change factor whereas a positive value indicates an increase in plant trait response.

To avoid pseudo-replication with regard to the effects of differences among different trait categories, we pooled the resulting multiple effect sizes (weighted by inverse variance) and corresponding variances per trait for studies providing different measures for the same traits of the same plant species (Leimu et al., 2006). Samples were pooled using the fixed-effects model (the *rma* function in the R package *metafor*), as we assumed a single true underlying effect size per trait of each plant species per study (Borenstein et al., 2009). The resulting 448 effect sizes and corresponding mean variances were termed ‘Data-I.’ This dataset was used to determine the responses of different functional traits to global environmental changes. Potential multiple effect sizes (different trait values) for one plant species per study in Data-I may lead to pseudo-replication when determining the response to global environmental changes at the functional group and plant species levels. To avoid this, we assumed a single true underlying effect size per plant species for each study and pooled the resulting multiple effect sizes (weighted by inverse variance) and corresponding variances per species of each study in Data-I using the same method. The new database including 218 effect sizes and corresponding mean variances was designated Data-II.

### Data Analysis

All meta-analytical calculations and statistical analyses were performed in R 3.1.3 (R Core Team, 2015) using the package *metafor* v1.9-7 (Viechtbauer, 2010). First, we used the Funnel plot and Egger’s regression to assess whether publication bias exists in our metadata sets (Data-I and Data-II). Funnel plots were plotted using the *funnel* function and visually inspected for the presence of asymmetry. Next, we tested the asymmetry of funnel plots using Egger’s test (Sterne and Egger, 2006). In this test, the standard normal deviate is regressed on precision (i.e., inverse of the standard error). The intercept in this regression corresponds to the slope in a weighted regression of the effect size on the standard error. This test can be performed using the *regtest* function included in the package *metafor*. Visual inspection and Egger’s test for asymmetry of the funnel plots showed that the results are not affected by publication bias (Data-I: *z* = 1.404, *p* = 0.160; Data-II: *z* = 0.550, *p* = 0.582; Supplementary Method S2).

To ascertain whether alien plants exhibit significant positive or negative responses to global environmental changes on average, we performed a general meta-analysis using a random-effects model on Data-II. To determine whether plant responses differ among the four global environmental change factors examined (precipitation, temperature, atmospheric CO_2_ concentration and N deposition), we constructed mixed-effects multivariate models using the *rma.mv* function. In this model, the global environmental factor was included as a fixed-effects moderator. To control for possible non-independence of effect sizes from studies including multiple alien plant species as well as single plant species used in multiple studies, we included study (i.e., publications from which data were extracted) and species identity as random factors in each model. To control for possible non-independence of effect sizes from species with shared evolutionary history, phylogenetic relatedness was included among the study species in the models (Supplementary Method S3).

We further determined whether the responses of the above six categories differed significantly according to global environmental changes using the same procedure as that for Data-I. The analysis was performed separately for each of the global environmental change components (changes in mean levels of precipitation, temperature, atmospheric CO_2_ levels or N deposition) using a subset of Data-I. Considering species variations in terms of life forms (woody and non-woody) and photosynthetic types (C3 and C4), we conducted further analyses to determine whether the effects of these four factors differ significantly across alien plants using Data-II. The same analysis was performed separately for each of the four factors using a Data-II subset.

In each model, we computed the weighted mean effect sizes and 95% confidence intervals (CI) for the moderator (i.e., components of global environmental change pooled together or treated individually). If the 95% CI around the mean did not include zero, the mean effect size estimate was considered significantly different from zero. The *Q*-test was applied (Koricheva et al., 2013; Scasta et al., 2016) to estimate differences in the mean effect sizes between woody and non-woody life forms or C3 and C4 photosynthetic types for the moderator. In these models, including a model structure (i.e., fixed-term), total heterogeneity (Q_T_) in effect sizes can be partitioned into heterogeneity explained by the model structure (*Q*_M_) and residual heterogeneity (*Q*_E_). We considered the fixed term significant when the model structure was assessed as significant.

## Results

**FIGURE 1:**
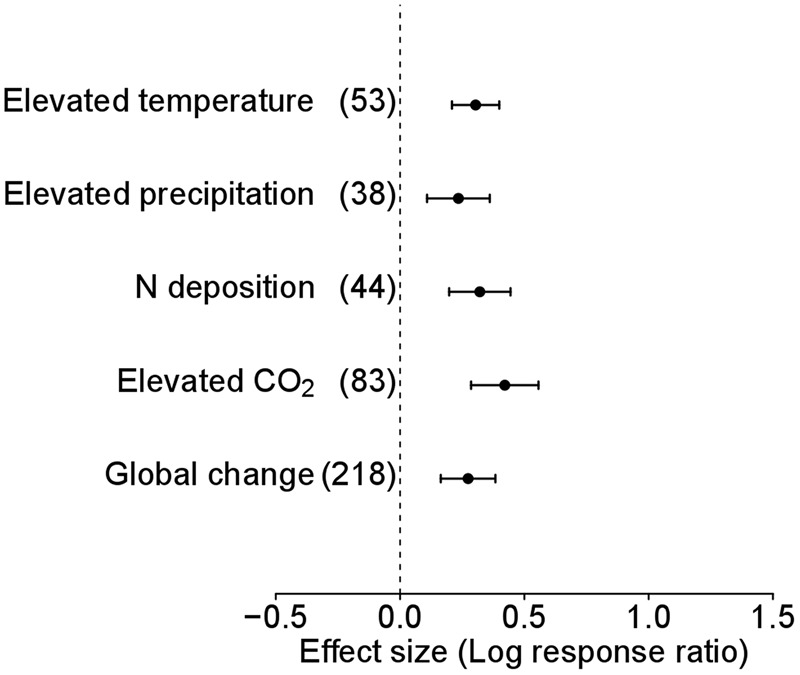
**Performance response (indicated by log response ratio of mean effect sizes) of alien plants to overall global environmental change, elevated temperature, elevated precipitation, N deposition and elevated CO_2_.** Error bars representing 95% confidence intervals around the mean effect size estimates were derived from the phylogenetically informed meta-analytic model. Sample sizes (i.e., number of studies) are provided in parentheses. The dashed vertical line indicates a zero effect of global environmental change components. In the case of precipitation, a number of studies imposed drought treatment. To facilitate comparisons with the other global change factors, we included studies that considered drought treatment as elevated precipitation by changing the control and treatment levels in the main analyses. For results of separate analyses of both drought and increased precipitation studies, please see Supplementary Figure S1.

To determine the response of plant species level and functional groups to global environmental changes, we pooled effect sizes (weighted by inverse variance) and corresponding variances per species of each study in Data-I to generate a new database, Data-II. In the analysis of Data-II without considering functional groups, alien plants showed overall significantly positive responses to environmental change (Ln*R* = 0.304, 95% CI = 0.209–0.399, *n* = 218; **Figure 1**) as well as each of the four global environmental change factors, i.e., elevated temperature (Ln*R* = 0.235, 95% CI = 0.108–0.361, *n* = 53; **Figure 1**), precipitation (Ln*R* = 0.321, 95% CI = 0.197–0.444, *n* = 38; **Figure 1**), N deposition (Ln*R* = 0.421, 95% CI = 0.286–0.557, *n* = 44; **Figure 1**), and atmospheric CO_2_ concentration (Ln*R* = 0.274, 95% CI = 0.164–0.384, *n* = 83; **Figure 1**).

**FIGURE 2:**
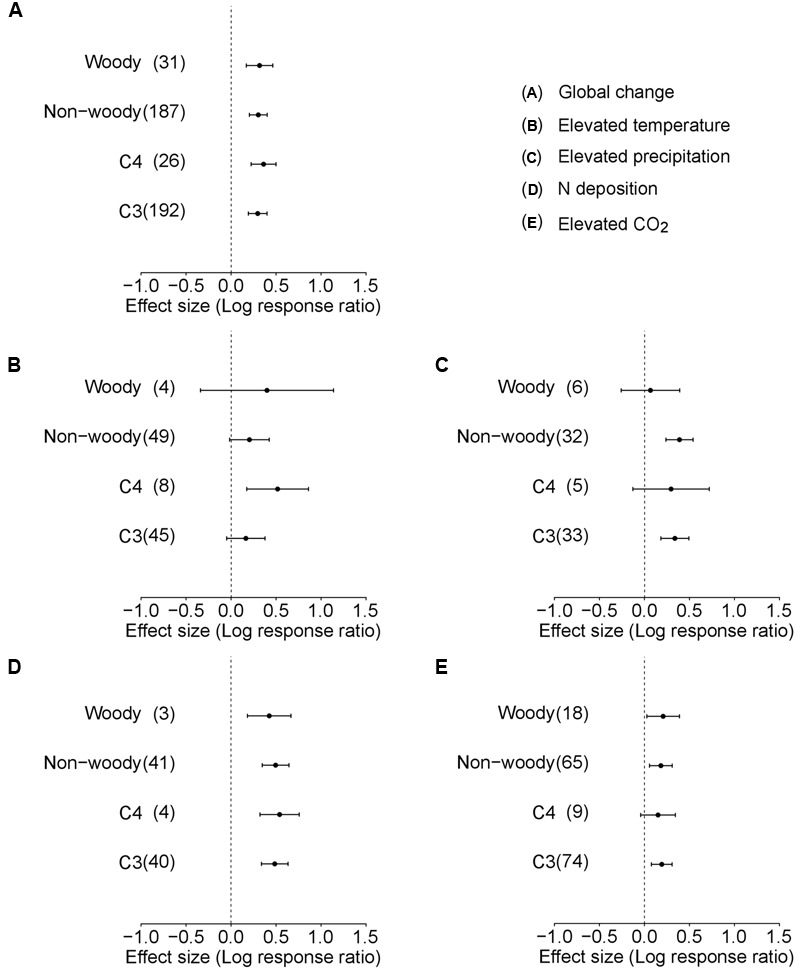
**Performance response (indicated by log response ratio of the mean effect sizes) of different life forms (woody and non-woody) and photosynthetic pathways (C3 and C4) of alien plants to overall global environmental change **(A)**, elevated temperature **(B)**, elevated precipitation **(C)**, N deposition **(D)**, and elevated CO_2_**(E)**.** Error bars representing 95% CIs around the mean effect size estimates were derived from the phylogenetically informed meta-analytic model. Sample sizes (i.e., number of studies) are provided in parentheses. The dashed vertical line indicates a zero effect of the global environmental change components. In the case of precipitation, a number of studies imposed drought treatment. To facilitate comparisons with other global change factors, we included studies that considered drought treatment as elevated precipitation by changing the control and treatment levels in the main analyses. For results of separate analyses of both drought and increased precipitation studies, please see Supplementary Figure S1.

In the analysis for Data-II considering life forms or photosynthetic types of species, the average plant responses were not affected by life forms (*Q*_M_ = 0.043, df = 1, *p* = 0.835; **Table 1**, **Figure 2**) or photosynthetic type (*Q*_M_ = 1.499, df = 1, *p* = 0.221; **Table 1**, **Figure 2**). In a separate analysis for individual environmental factors, no significant differences were observed between woody and non-woody or C3 and C4 alien plants in response to elevated precipitation, N deposition, and atmospheric CO_2_ concentration (**Table 1**, **Figure 2**). However, C4 alien plants exhibited a more positive response to elevated temperature than C3 alien plants (*Q*_M_ = 0.266, df = 1, *p* = 0.034; **Table 1**; **Figure 2**).

**Table 1:** Results of a phylogenetically informed meta-analysis comparing the responses of C3 and C4 alien plants or non-woody and woody alien plants to environmental changes (i.e., mean levels of precipitation, temperature, atmospheric CO_2_ concentration or nitrogen deposition).

				Effect size			Random effects (Variance component)			Q-test		
			Sample size	Mean	Lower 95% CI	Upper 95% CI	Species	Phylogeny	Study	QM	df	*P*
Global change	Photosynthetic pathway	C3	192	0.2949	0.191	0.3987	0	0.0041	0.1218	1.4985	1	0.2209
		C4	26	0.3601	0.2218	0.4983						
	Lifeform	Non-woody	187	0.3014	0.2027	0.4001	0	0.003	0.1243	0.0432	1	0.8354
		Woody	31	0.3155	0.1684	0.4625						
Precipitation	Photosynthetic pathway	C3	33	0.3362	0.1805	0.4919	0.0075	0.0001	0.1176	0.0335	1	0.8548
		C4	5	0.294	-0.1301	0.7181						
	Lifeform	Non-woody	32	0.3875	0.2367	0.5382	0.0069	0	0.0999	3.1145	1	0.0776
		Woody	6	0.0649	-0.2601	0.3899						
Warming	Photosynthetic pathway	C3	45	0.1628	-0.0501	0.3756	0.0052	0	0.2659	4.5131	1	0.0336
		C4	8	0.5157	0.1729	0.8585						
	Lifeform	Non-woody	49	0.2026	-0.0173	0.4225	0.0094	0	0.2748	0.246	1	0.6199
		Woody	4	0.3977	-0.3411	1.1365						
CO_2_	Photosynthetic pathway	C3	74	0.1922	0.0771	0.3073	0	0.006	0.0393	0.2234	1	0.6365
		C4	9	0.1507	-0.0419	0.3432						
	Lifeform	Non-woody	65	0.1823	0.0561	0.3084	0	0.0073	0.0409	0.0882	1	0.7664
		Woody	18	0.2077	0.0265	0.3889						
N deposition	Photosynthetic pathway	C3	40	0.4865	0.3391	0.634	0	0	0.1161	0.3909	1	0.5318
		C4	4	0.5404	0.3225	0.7583						
	Lifeform	Non-woody	41	0.496	0.3476	0.6445	0	0	0.1155	0.4264	1	0.5138
		Woody	3	0.425	0.1832	0.6669						

*The analysis was performed for all four components of environmental change combined (i.e., global change) and individually for each component. The *Q*-test statistic and associated *P-*value were used to test for differences between functional groups. In the case of precipitation, a number of studies imposed drought treatment. To facilitate comparisons with the other global change factors, we included these studies that considered drought treatment as elevated precipitation by changing the control and treatment levels in the main analyses. Results of separate analyses of both drought and increased precipitation studies are presented in Supplementary Figure S1.*

**FIGURE 3:**
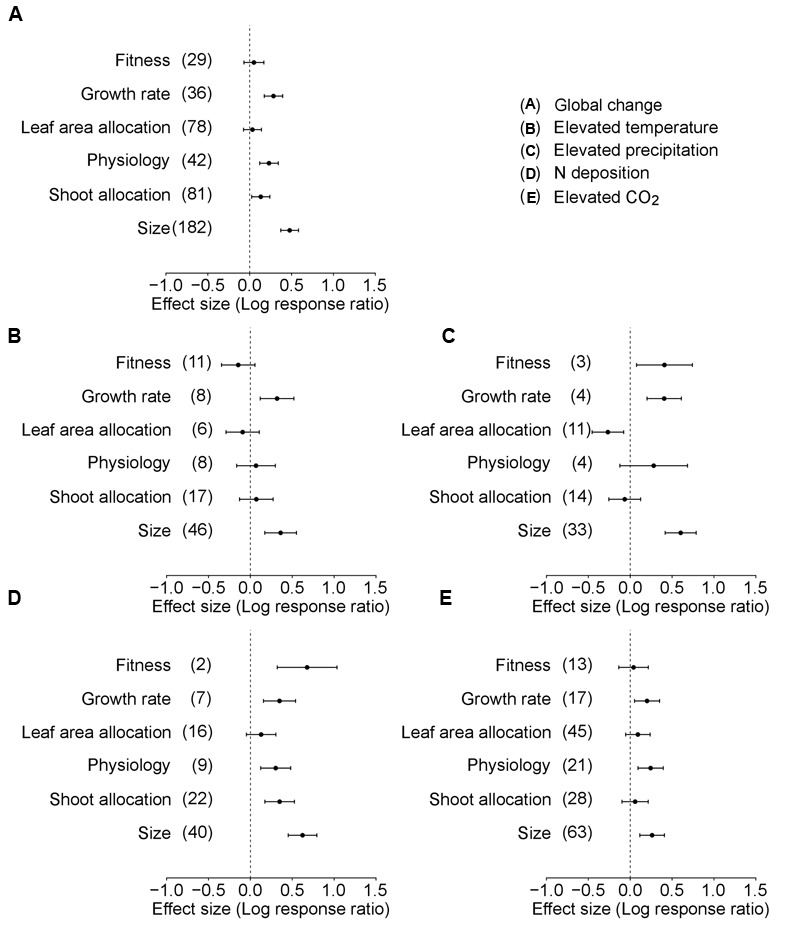
**Performance response (indicated by log response ratio of mean effect sizes) of different trait categories of alien plants to overall global environmental change **(A)**, elevated temperature **(B)**, elevated precipitation **(C)**, N deposition **(D)**, and elevated CO_2_**(E)**.** Error bars representing 95% CIs around the mean effect size estimates were derived from the phylogenetically informed meta-analytic model. Sample sizes (i.e., number of studies) are provided in parentheses. The dashed vertical line indicates a zero effect of the global environmental change components. In the case of precipitation, a number of studies imposed drought treatment. To facilitate comparisons with the other global change factors, we included studies that considered drought treatment as elevated precipitation by changing the control and treatment levels in the main analyses. For results of separate analyses of both drought and increased precipitation studies, please see Supplementary Figure S1.

In the analysis of Data-I considering the differences among six trait categories, global environmental changes had significant positive effects on plant growth rate, physiology, shoot allocation, and size (**Table 2**, **Figure 3**). However, the patterns were different for each global environmental change factor. Warming induced significant positive responses in plant growth rate and size (**Table 2**, **Figure 3B**), increased precipitation affected plant fitness, growth rate and size (**Table 2**, **Figure 3C**), increased N deposition influenced plant fitness, growth rate, physiology, shoot allocation, and size (**Table 2**, **Figure 3D**), and CO_2_ enrichment promoted plant growth rate, physiology, and size (**Table 2**, **Figure 3E**).

**Table 2:** Results of a phylogenetically informed meta-analysis to determine the responses of six trait categories to environmental changes (i.e., mean levels of precipitation, temperature, atmospheric CO_2_ concentration or nitrogen deposition).

		Sample size	Effect size			Random effects (Variance component)		
			Mean	Lower 95% CI	Upper 95% CI	Species	Phylogeny	Study
Global change	Fitness	29	0.0506	-0.0685	0.1697	0.0217	0.0052	0.0859
	Growth rate	36	0.2842	0.1745	0.3938			
	Leaf area allocation	78	0.0333	-0.0737	0.1403			
	Physiology	42	0.2305	0.1199	0.3412			
	Shoot allocation	81	0.1323	0.0238	0.2408			
	Size	182	0.4773	0.3714	0.5832			
Precipitation	Fitness	3	0.4093	0.0756	0.7429	0.0151	0.0076	0.1362
	Growth rate	4	0.4059	0.2012	0.6106			
	Leaf area allocation	11	-0.2661	-0.4547	-0.0775			
	Physiology	4	0.2806	-0.1232	0.6845			
	Shoot allocation	14	-0.0651	-0.2547	0.1244			
	Size	33	0.6028	0.4175	0.7880			
Warming	Fitness	11	-0.1448	-0.3446	0.0550	0.0450	0.0000	0.2000
	Growth rate	8	0.3188	0.1178	0.5198			
	Leaf area allocation	6	-0.0923	-0.2905	0.1060			
	Physiology	8	0.0673	-0.1640	0.2985			
	Shoot allocation	17	0.0711	-0.1292	0.2715			
	Size	46	0.3609	0.1719	0.5499			
CO_2_	Fitness	13	0.0400	-0.1362	0.2161	0.0119	0.0130	0.0281
	Growth rate	17	0.2010	0.0516	0.3504			
	Leaf area allocation	45	0.0917	-0.0545	0.2380			
	Physiology	21	0.2439	0.0931	0.3947			
	Shoot allocation	28	0.0585	-0.0981	0.2152			
	Size	63	0.2615	0.1147	0.4083			
N deposition	Fitness	2	0.6764	0.3195	1.0332	0.0019	0.0127	0.0527
	Growth rate	7	0.3468	0.1550	0.5385			
	Leaf area allocation	16	0.1272	-0.0484	0.3029			
	Physiology	9	0.3010	0.1217	0.4804			
	Shoot allocation	22	0.3484	0.1722	0.5247			
	Size	40	0.6218	0.4506	0.7930			

*The analysis was performed for all four components of environmental change combined (i.e., global change) and individually for each component. In the case of precipitation, a number of studies imposed a drought treatment. To facilitate comparisons with the other global change factors, we included studies that considered drought treatment as elevated precipitation by changing the control and treatment levels in the main analyses. The results of separate analyses of both drought and increased precipitation studies are presented in Supplementary Figure S1.*

## Discussion

Alien plants are proposed to benefit from global environmental changes. Data from the current phylogenetically controlled meta-analysis suggested that alien plants show a positive plastic response in performance to the four environmental change components assessed, and furthermore, are not dependent on life forms and photosynthetic types. Notably, however, C4 plants showed a more positive response to warming than C3 plants. Our findings provide further evidence that all four global environmental change components promote the growth of alien plants mainly by increasing growth rate and size, although the patterns of effects on different alien plant traits vary among these components.

The finding of positive plastic responses to components of environmental change by alien plants is in line with data from our recent meta-analysis comparing the growth performance response to these four factors between invasive alien and native plants (Liu et al. unpublished). Recently, Sorte et al. (2013) published a meta-analysis on the responses of alien and native organisms to climate change and showed that terrestrial organisms show a positive performance response to CO_2_ enrichment and elevated precipitation. However, warming had no significant effects. One possible explanation for the discrepancies between the results is that Sorte et al. (2013) evaluated the responses of both plants and animals to environmental change and did not correct for phylogenetic non-independence of the species analyzed (Chamberlain et al., 2012). Furthermore, we found that C4 plants responded more positively than C3 plants under warming conditions. This could be explained by the fact that unlike C3 plants, C4 plants generally originate from warm climates (Sage and Monson, 1998; Lara and Andreo, 2011) and the plastic ability of photosynthesis is also greater for C4 than C3 plants (Yamori et al., 2014). Moreover, C4 plants show better nitrogen and water efficiency than C3 plants at high temperatures (Yamori et al., 2014). Although C4 plants are significantly more efficient in using CO_2_, we observed similar responses in both C4 and C3 plants under CO_2_ enrichment, which may be attributable to the fact that CO_2_ enrichment enhances the growth of C4 plants specifically under drought stress conditions (Xu et al., 2014).

Global environmental changes have significant positive effects on alien plant growth rate and size, as revealed by both joint and separate analyses. In our experiments, alien plants displayed increased growth rates and sizes in response to the four factors analyzed. High growth rate is frequently positively associated with high annual biomass production (trait category “size”) (van Kleunen et al., 2010). As expected, we observed consistency in responses between the growth rate and size categories. Generally, alien plant species tolerate a broad range of environmental conditions (Vilà et al., 2007; Hellmann et al., 2008) and display higher potential growth rates and sizes than native plant species under current global environmental conditions (van Kleunen et al., 2010; Davidson et al., 2011), which may explain why some alien plants outperform native plants. The global environmental change-induced positive responses of both trait categories of alien plants may become more advantageous, thereby facilitating their growth to a higher extent than that of native plants in the future. Moreover, specific factors, such as elevated precipitation and increased N deposition, could increase the fitness of alien plants, further contributing to their success under global environmental change conditions.

Global environmental changes had a significant positive effect on shoot allocation of alien plants in the joint analysis. However, only increased N deposition exerted a significant positive effect in the separate analysis, consistent with data from earlier studies (Poorter and Nagel, 2000; Drenovsky et al., 2012; Poorter et al., 2012; Freschet et al., 2015). The functional equilibrium hypothesis (Brouwer, 1962, 1963) suggests that plants shift their allocation toward roots when below-ground resources are low. Generally, plants are limited in productivity by nutrient availability, particularly N and/or P (Vitousek and Howarth, 1991; Fisher et al., 2012). Increased N deposition could change the resource limitation of alien plants from below- to above-ground, leading to a shift in allocation to shoots. Compared with other global environmental change factors, only increased N deposition exerted significant positive effects on all trait categories, except leaf area allocation. This finding may be attributable to the invasive nature of most of the alien species used in the analysis. According to Dostal et al. (2013), alien plants introduced from more N-rich habitats are more invasive and thus more likely to adapt to environments with high N levels.

Global environmental changes had a significant positive effect on the physiology of alien plants in the joint analysis. However, only increased N deposition and CO_2_ enrichment exerted significant positive effects in the separate analysis. While elevated temperature and precipitation positively affected the physiology of alien plants, the effects were not significant. Our results contradict those of a recent meta-analysis of 197 studies by Liang et al. (2013), which showed that warming has overall positive effects on plant photosynthesis. Generally, most plants adjust their photosynthetic characteristics to temperature acclimation and the photosynthesis-temperature curve is often symmetrical or bell-shaped (e.g., Yamori et al., 2010, 2014). The discrepancy between our findings and those of other studies may be attributed to the different warming treatments among experiments and temperature sensitivities of various plant species (Llorens et al., 2004). Furthermore, water availability is an important factor limiting plant physiology, and many studies have shown that plant species have higher photosynthetic rates under increased than ambient precipitation. The non-significant effect in this meta-analysis is most likely attributable to the low statistical power.

To facilitate comparisons with other global change factors in the present meta-analysis, we included a number of studies that considered drought treatment as elevated precipitation by changing the control and treatment levels. However, precipitation levels are variable among different regions. For example, precipitation patterns associated with global changes are predicted to vary, with some areas receiving more precipitation than the others in the US (Naz et al., 2016). Therefore, we performed *post hoc* analyses for the different subsets considering whether water availability in a study increased or decreased, compared to the ambient levels. We observed opposite effects of increased and decreased precipitation on alien plants. Specifically, increased precipitation promoted growth whereas decreased precipitation inhibited growth (Supplementary Figure S1), suggesting that invasiveness of alien plants decreases when the climate becomes drier.

Many alien plants do not simply disperse on their own, but are spread by humans or as a result of changing environments (White et al., 2013; Scasta et al., 2015). The present meta-analysis has provided a quantitative summary of data showing that global environmental changes create favorable environmental conditions that could promote the growth of alien plants. Thus, alien plants have greater ecological effects and cause potential damage to ecosystem functions and services with global environmental changes (Kettenring and Adams, 2011; Vila et al., 2011; Scasta et al., 2015). However, since only 24 of the 111 total studies included in our meta-analysis manipulated more than one global environment change factor, we summarized the patterns of alien plant responses to individual components. Many of these components change simultaneously, and combination of more than one may exert different effects on plant performance related to a single component (Dukes et al., 2005; Xu and Zhou, 2006; Bloor et al., 2010; Dieleman et al., 2012). Further large-scale empirical studies are essential to determine the interactive effects of environmental change components on alien plant performance.

## Conclusion

Data from our phylogenetically controlled meta-analysis showed that elevated temperature, precipitation, N deposition, and atmospheric CO_2_ concentration promote alien plant growth. Elevated temperature had a more positive effect on C4 than C3 plants, indicating that C4 alien plants have higher potential for invasion than C3 plants under future global warming conditions. While different global environmental change factors affected distinct functional traits of alien plants, all four components promoted growth predominantly by increasing growth rate and size, potentially representing one of the mechanisms used by alien plants to adapt and facilitate successful growth under ever-changing environmental conditions.

## Author Contributions

YL designed this study. YL, JJ, and ZD compiled the data. YL and JJ analyzed the data. JJ drafted the manuscript with major inputs from YL as well as contributions from ZD and FL.

## Conflict of Interest Statement

The authors declare that the research was conducted in the absence of any commercial or financial relationships that could be construed as a potential conflict of interest.

## Footnotes

1 http://apps.webofknowledge.com/

2 www.cnki.net
