# Supplementary material for: How Will Global Environmental Changes Affect the Growth of Alien Plants?
Source: Front Plant Sci. 2016 Nov 1;7:1623. doi: 10.3389/fpls.2016.01623 (PMC5088532; doi:10.3389/fpls.2016.01623)
Supplement: Supplementary file 1 [file Data_Sheet_1.PDF]

Supplementary materials

Method S1 List of published studies from which data were extracted

Allen EB. 1982. Germination and competition of Salsola kali with native C3 and C4 species under three temperature regimes. *Bulletin of the Torrey Botanical Club*: 39-46.

Anderson LJ, Cipollini D. 2013. Gas exchange, growth, and defense responses of invasive *Alliaria petiolata* (brassicaceae) and native *Geum vernum* (rosaceae) to elevated atmospheric CO_2 and warm spring temperatures. *American Journal of Botany* 100(8): 1544-1554.

Anderson LJ, Maherali H, Johnson HB, Polley HW, Jackson RB. 2001. Gas exchange and photosynthetic acclimation over subambient to elevated CO_2 in a C3-C4 grassland. *Global Change Biology* 7(6): 693-707.

Bakker J, Wilson S. 2001. Competitive abilities of introduced and native grasses. *Plant Ecology* 157(2): 119-127.

Ban Z, Wang Q. 2015. Responses of the competition between *Alternanthera philoxeroides* and *Sambucus chinensis* to simulated warming. *Chinese Journal of Plant Ecology* 39(1): 43-51.

Baruch Z, Jackson RB. 2005. Responses of tropical native and invader C4 grasses to water stress, clipping and increased atmospheric CO_2 concentration. *Oecologia* 145(4): 522-532.

Belote RT, Weltzin JF, Norby RJ. 2004. Response of an understory plant community to elevated CO_2 depends on differential responses of dominant invasive species and is mediated by soil water availability. *New Phytologist* 161(3): 827-835.

Blank RR. 2004. Effects of CO_2 enrichment on plant-soil relationships of *Lepidium latifolium*. *Plant and Soil* 262(1-2): 159-167.

Blicker PS, Olson BE, Wraith JM. 2003. Water use and water-use efficiency of the invasive *Centaurea maculosa* and three native grasses. *Plant and Soil* 254(2): 371-381.

Blumenthal D, Chimner RA, Welker JM, Morgan JA. 2008. Increased snow facilitates plant invasion in mixedgrass prairie. *New Phytologist* 179(2): 440-448.

Blumenthal DM, Resco V, Morgan JA, Williams DG, Lecain DR, Hardy EM, Pendall E, Bladyka E. 2013. Invasive forb benefits from water savings by native plants and carbon fertilization under elevated CO_2 and warming. *New Phytologist* 200(4): 1156-1165.

Bradford MA, Schumacher HB, Catovsky S, Eggers T, Newingtion JE, Tordoff GM. 2007. Impacts of invasive plant species on riparian plant assemblages: interactions with

elevated atmospheric carbon dioxide and nitrogen deposition. *Oecologia* 152(4): 791-803.

Brock MT, Galen C. 2005. Drought tolerance in the alpine dandelion, *Taraxacum ceratophorum* (Asteraceae), its exotic congener *T. officinale*, and interspecific hybrids under natural and experimental conditions. *American Journal of Botany* 92(8): 1311-1321.

Chai WL, Lei YB, Li YP, Xiao HF, Feng YL. 2014. Responses of invasive *Chromolaena odorata* and native *Eupatorium heterophyllum* to atmospheric CO_2 enrichment. *Acta Ecologica Sinica*,34(13): 3744-3751.

Chuine I, Morin X, Sonié L, Collin C, Fabreguettes J, Degueldre D, Salager JL, Roy J. 2012. Climate change might increase the invasion potential of the alien C4 grass *Setaria parviflora* (Poaceae) in the Mediterranean Basin. *Diversity and Distributions* 18(7): 661-672.

Concilio AL, Loik ME. 2013. Elevated nitrogen effects on Bromus tectorum dominance and native plant diversity in an arid montane ecosystem. *Applied Vegetation Science* 16(4): 598-609.

Cox JA, Conran JG. 1996. The effect of water stress on the life cycles of *Erodium crinitum* Carolin and *Erodium cicutarium* (L.) L'Hérit. ex Alton (Geraniaceae). *Australian Journal of Ecology* 21(2): 235-240.

Cuda J, Skalova H, Janovsky Z, Pysek P. 2015. Competition among native and invasive Impatiens species: the roles of environmental factors, population density and life stage. *AoB Plants* 7.

Dalmolin AC, Dalmagro HJ, Lobo FdA, Antunes MZ, Jr., Ortiz CER, Vourlitis GL. 2013. Photosynthetic light and carbon dioxide response of the invasive tree, Vochysia divergens Pohl, to experimental flooding and shading. *Photosynthetica* 51(3): 379-386.

Diaz R, Manrique V, He Z, Overholt WA. 2012. Effect of elevated CO_2 on tropical soda apple and its biological control agent *Gratiana boliviana* (Coleoptera: Chrysomelidae). *Biocontrol Science and Technology* 22(7): 763-776.

Domènech R, Vilà M. 2008. Response of the invader *Cortaderia selloana* and two coexisting natives to competition and water stress. *Biological Invasions* 10(6): 903-912.

Dukes JS. 2002. Comparison of the effect of elevated CO2 on an invasive species (*Centaurea solstitialis*) in monoculture and community settings. *Plant Ecology* 160(2): 225-234.

Dukes JS, Chiariello NR, Loarie SR, Field CB. 2011. Strong response of an invasive plant species (*Centaurea solstitialis* L.) to global environmental changes. *Ecological Applications* 21(6): 1887-1894.

Eller F, Lambertini C, Loc Xuan N, Brix H. 2014. Increased invasive potential of non-native *Phragmites australis*: elevated CO_2 and temperature alleviate salinity effects on photosynthesis and growth. *Global Change Biology* 20(2): 531-543.

Eskelinen A, Harrison S. 2014. Exotic plant invasions under enhanced rainfall are constrained by soil nutrients and competition. *Ecology* 95(3): 682-692.

Funk JL, Zachary VA. 2010. Physiological responses to short-term water and light stress in native and invasive plant species in southern California. *Biological Invasions* 12(6): 1685-1694.

Ge JL, He JQ, Sun XF, Chen Q. 2010. Ecological Responses of the Invasive Alien Plant *Solidago canadensis* to Changes of Soil Water Content. *Acta Botanica Boreali-Occidentalia Sinica* 30 (03): 575-585.

Geng Y-P, Pan X-Y, Xu C-Y, Zhang W-J, Li B, Chen J-K. 2006. Phenotypic plasticity of invasive *Alternanthera philoxeroides* in relation to different water availability, compared to its native congener. *Acta oecologica* 30(3): 380-385.

Gomez-Casanovas N, Blanc-Betes E, Gonzalez-Meler MA, Azcon-Bieto J. 2007. Changes in respiratory mitochondrial machinery and cytochrome and alternative pathway activities in response to energy demand underlie the acclimation of respiration to elevated CO_2 in the invasive Opuntia ficus-indica. *Plant Physiology* 145(1): 49-61.

Grossman JD, Rice KJ. 2014. Contemporary evolution of an invasive grass in response to elevated atmospheric CO_2 at a Mojave Desert FACE site. *Ecology letters* 17(6): 710-716.

Hättenschwiler S, Körner C. 2003. Does elevated CO2 facilitate naturalization of the non-indigenous *Prunus laurocerasus* in Swiss temperate forests? *Functional Ecology* 17(6): 778-785.

He W-M, Li J-J, Peng P-H. 2012. A congeneric comparison shows that experimental warming enhances the growth of invasive *Eupatorium adenophorum*. *PloS One* 7(4).

He WM, Yu GL, Sun ZK. 2011. Nitrogen deposition enhances *Bromus tectorum* invasion: biogeographic differences in growth and competitive ability between China and North America. *Ecography* 34(6): 1059-1066.

Hely SEL. 2008. The responses of C4 invasive grass *Eragrostis curvula* and C3 native Australian grass *Austrodanthonia racemosa* under elevated CO_2 and water limitation. University of New South Wales.

Horton JL, Clark JL. 2001. Water table decline alters growth and survival of *Salix gooddingii* and *Tamarix chinensis* seedlings. *Forest Ecology and Management* 140(2): 239-247.

Hoya A, Shibaike H, Morita T, Ito M. 2004. Germination and seedling survivorship

characteristics of hybrids between native and alien species of dandelion (Taraxacum). *Plant Species Biology* 19(2): 81-90.

Hussner A, Hofstra D, Jahns P, Clayton J. 2015. Response capacity to CO_2 depletion rather than temperature and light effects explain the growth success of three alien Hydrocharitaceae compared with native Myriophyllum triphyllum in New Zealand. *Aquatic Botany* 120: 205-211.

Hussner A, Jahns P. 2014. European native Myriophyllum spicatum showed a higher HCO_3^- use capacity than alien invasive *Myriophyllum heterophyllum*. *Hydrobiologia* 746(1): 171-182.

Huxman TE, Hamerlynck EP, Jordan DN, Salsman KJ, Smith SD. 1998. The effects of parental CO_2 environment on seed quality and subsequent seedling performance in Bromusrubens. *Oecologia* 114(2): 202-208.

Huxman TE, Smith SD. 2001. Photosynthesis in an invasive grass and native forb at elevated CO_2 during an El Nino year in the Mojave Desert. *Oecologia* 128(2): 193-201.

Hwang BC, Lauenroth WK. 2008. Effect of nitrogen, water and neighbor density on the growth of *Hesperis matronalis* and two native perennials. *Biological Invasions* 10(5): 771-779.

Hwang BC, Lauenroth WK. 2010. Effect of nitrogen, water and neighbors on the growth of *Hesperis matronalis* in a natural community. *American Midland Naturalist* 163(1): 212-219.

Ignace DD, Huxman TE, Weltzin JF, Williams DG. 2007. Leaf gas exchange and water status responses of a native and non-native grass to precipitation across contrasting soil surfaces in the Sonoran Desert. *Oecologia* 152(3): 401-413.

Jamieson MA, Quintero C, Blumenthal DM. 2013. Interactive effects of simulated nitrogen deposition and altered precipitation patterns on plant allelochemical concentrations. *Journal of Chemical Ecology* 39(9): 1204-1208.

Kan LY, Xie GS, Wang JK. 2009. Effect of drought stress on the growth and eco-physiologic characteristics of invasive plant *Eupatorium catarium* seedlings. *Chinese Journal of Tropical Crops* 30(05): 608-612.

Kao-Kniffin J, Freyre DS, Balser TC. 2011. Increased methane emissions from an invasive wetland plant under elevated carbon dioxide levels. *Applied Soil Ecology* 48(3): 309-312.

Lei Y-b, Wang W-b, Feng Y-l, Zheng Y-l. 2012. Synergistic interactions of CO_2 enrichment and nitrogen deposition promote growth and ecophysiological advantages of invading *Eupatorium adenophorum* in Southwest China. *Planta* 236(4): 1205-1213.

Lei YB, Feng YL, Zheng YL, Wang RF, Gong HD, Zhang YP. 2011. Innate and evolutionarily

increased advantages of invasive *Eupatorium adenophorum* over native *E. japonicum* under ambient and doubled atmospheric CO_2 concentrations. *Biological Invasions* 13(12): 2703-2714.

Leskovšek R, Eler K, Batič F, Simončič A. 2012. Water and nitrogen use efficiency of common ragweed (*Ambrosia artemisiifolia* L.) at different nitrogen and water levels. *Acta agriculturae Slovenica* 99(1): 41-47.

Li H-L, Ning L, Alpert P, Li J-M, Yu F-H. 2014. Responses to simulated nitrogen deposition in invasive and native or non-invasive clonal plants in China. *Plant Ecology* 215(12): 1483-1492.

Limpens J, Berendse F, Klees H. 2003. N deposition affects N availability in interstitial water, growth of Sphagnum and invasion of vascular plants in bog vegetation. *New Phytologist* 157(2): 339-347.

Liu Q. 2012. Effects of simulated warming and nitrogen deposition on growth and morphological characteristics of several species of invasive Asteraceae plants. Master thesis, Chengdu University of Technology.

Luo YJ, Guo WH, Yuan YF, Liu J, Du N, Wang RQ. 2014. Increased nitrogen deposition alleviated the competitive effects of the introduced invasive plant Robinia pseudoacacia on the native tree Quercus acutissima. *Plant and Soil* 385(1-2): 63-75.

Manea A, Leishman MR. 2015. Competitive interactions between established grasses and woody plant seedlings under elevated CO_2 levels are mediated by soil water availability. *Oecologia* 177(2): 499-506.

Manea A, Leishman MR, Downey PO. 2011. Exotic C4 grasses have increased tolerance to glyphosate under elevated carbon dioxide. *Weed Science* 59(1): 28-36.

Maron JL, Marler M. 2008. Field-based competitive impacts between invaders and natives at varying resource supply. *Journal of Ecology* 96(6): 1187-1197.

Mateos-Naranjo E, Redondo-Gomez S, Alvarez R, Cambrolle J, Gandullo J, Enrique Figueroa M. 2010. Synergic effect of salinity and CO_2 enrichment on growth and photosynthetic responses of the invasive cordgrass *Spartina densiflora*. *Journal of Experimental Botany* 61(6): 1643-1654.

Mozdzer TJ, Megonigal JP. 2012. Jack-and-master trait responses to elevated CO_2 and N: a comparison of native and introduced *Phragmites australis*. *PloS One* 7(10): e42794.

Nagel JM, Huxman TE, Griffin KL, Smith SD. 2004. CO_2 enrichment reduces the energetic cost of biomass construction in an invasive desert grass. *Ecology* 85(1): 100-106.

Nernberg D, Dale MRT. 1997. Competition of five native prairie grasses with Bromus inermis

under three moisture regimes. *Canadian Journal of Botany* 75(12): 2140-2145.

Ni G, Zhao P, Wu W, Lu XK, Zhao XH, Zhu LW, Niu JF. 2014. A hybrid of the invasive plant *Sphagneticola trilobata* has similar competitive ability but different response to nitrogen deposition compared to parent. *Ecological Research* 29(2): 331-339.

Osone Y, Yazaki K, Masaki T, Ishida A. 2014. Responses to nitrogen pulses and growth under low nitrogen availability in invasive and native tree species with differing successional status. *J Plant Res* 127(2): 315-328.

Patterson DT. 1993. Effects of day and night temperature on goatsrue (*Galega officinalis*) and alfalfa (*Medicago sativa*) growth. *Weed Science*: 38-45.

Qaderi M, Reid D. 2008. Combined effects of temperature and carbon dioxide on plant growth and subsequent seed germinability of *Silene noctiflora*. *International Journal of Plant Sciences* 169(9): 1200-1209.

Qaderi MM, Lynch AL, Godin VJ, Reid DM. 2013. Single and interactive effects of temperature, carbon dioxide, and watering regime on the invasive weed black knapweed (*Centaurea nigra*). *Ecoscience* 20(4): 328-338.

Qi SY, Guo XH, Zhao M. 2009. Effects of elevated carbon dioxide and ozone concentrations on leaf morphological characteristics of invasive plant *Galinsona parviflora*. *Journal of Northeast Normal University (Natural Science Edition)* 41 (2): 149-153.

Qing H, Yao Y, Xiao Y, Hu F, Sun Y, Zhou C, An S. 2011. Invasive and native tall forms of *Spartina alterniflora* respond differently to nitrogen availability. *Acta oecologica* 37(1): 23-30.

Quan GM, Mao DJ, Zhang JE, Xie JF, Xu HQ, An M. 2015. Response of invasive *Chromolaena odorata* and two coexisting weeds to contrasting irradiance and nitrogen. *Photosynthetica*: 1-11.

Rahlao SJ, Esler KJ, Milton SJ, Barnard P. 2010. Nutrient addition and moisture promote the invasiveness of crimson fountaingrass (*Pennisetum setaceum*). *Weed Science* 58(2): 154-159.

Raizada P, Singh A, Raghubanshi A. 2009. Comparative response of seedlings of selected native dry tropical and alien invasive species to CO_2 enrichment. *Journal of Plant Ecology* 2(2): 69-75.

Rao LE, Allen EB. 2010. Combined effects of precipitation and nitrogen deposition on native and invasive winter annual production in California deserts. *Oecologia* 162(4): 1035-1046.

Reynolds LV, Cooper DJ. 2010. Environmental tolerance of an invasive riparian tree and its

potential for continued spread in the southwestern US. *Journal of Vegetation Science* 21(4): 733-743.

Sasek TW, Strain BR. 1991. Effects of CO_2 enrichment on the growth and morphology of a native and an introduced honeysuckle vine. *American Journal of Botany*: 69-75.

Shabbir A, Dhileepan K, Khan N, Adkins S. 2014. Weed–pathogen interactions and elevated CO_2: growth changes in favour of the biological control agent. *Weed Research* 54(3): 217-222.

Sheppard CS, Alexander JM, Billeter R. 2012. The invasion of plant communities following extreme weather events under ambient and elevated temperature. *Plant Ecology* 213(8): 1289-1301.

Sheppard CS, Stanley MC. 2014. Does elevated temperature and doubled CO_2 increase growth of three potentially invasive plants? *Invasive Plant Science and Management* 7(2):237-246.

Sigueenza C, Corkidi L, Allen EB. 2006. Feedbacks of soil inoculum of mycorrhizal fungi altered by N deposition on the growth of a native shrub and an invasive annual grass. *Plant and Soil* 286(1-2): 153-165.

Singh H, Menka, Srivastava P, Singh V. 2011. Effect of nitrogen addition on the architecture and biomass allocation of two invasive plant species (*Ageratum conyzoides* L. and *Parthenium hysterophorus* L.). *Proceedings of the National Academy of Sciences India Section B-Biological Sciences* 81: 348-358.

Smith SD, Huxman TE, Zitzer SF, Charlet TN, Housman DC, Coleman JS, Fenstermaker LK, Seemann JR, Nowak RS. 2000. Elevated CO_2 increases productivity and invasive species success in an arid ecosystem. *Nature* 408(6808): 79-82.

Song L-Y, Li C-H, Peng S-L. 2010. Elevated CO_2 increases energy-use efficiency of invasive *Wedelia trilobata* over its indigenous congener. *Biological Invasions* 12(5): 1221-1230.

Song L, Wu J, Li C, Li F, Peng S, Chen B. 2009. Different responses of invasive and native species to elevated CO_2 concentration. *Acta Oecologica-International Journal of Ecology* 35(1): 128-135.

Steiner JJ, Brewer TG, Griffith SM. 2001. Temperature effects on interspecific interference among two native wetland grasses and tall fescue. *Agronomy Journal* 93(5): 1020-1027.

Sullivan L, Wildova R, Goldberg D, Vogel C. 2010. Growth of three cattail (Typha) taxa in response to elevated CO_2. *Plant Ecology* 207(1): 121-129.

Sun Y, Ding J, Frye M. 2010. Effects of resource availability on tolerance of herbivory in the invasive *Alternanthera philoxeroides* and the native *Alternanthera sessilis*. *Weed*

Research 50(6): 527-536.

Thomsen MA, Corbin JD, D'Antonio CM. 2006. The effect of soil nitrogen on competition between native and exotic perennial grasses from northern coastal California. *Plant Ecology* 186(1): 23-35.

Tomassen HBM, Smolders AJP, Limpens J, Lamers LPM, Roelofs JGM. 2004. Expansion of invasive species on ombrotrophic bogs: desiccation or high N deposition? *Journal of Applied Ecology* 41(1): 139-150.

Tooth IM, Leishman MR. 2013. Post-fire resprouting responses of native and exotic grasses from Cumberland Plain Woodland (Sydney, Australia) under elevated carbon dioxide. *Austral Ecology* 38(1): 1-10.

Tooth IM, Leishman MR. 2014. Elevated carbon dioxide and fire reduce biomass of native grass species when grown in competition with invasive exotic grasses in a savanna experimental system. *Biological Invasions* 16(2): 257-268.

Tyler AC, Lambrinos JG, Grosholz ED. 2007. Nitrogen inputs promote the spread of an invasive marsh grass. *Ecological Applications* 17(7): 1886-1898.

Vallano DM, Selmants PC, Zavaleta ES. 2012. Simulated nitrogen deposition enhances the performance of an exotic grass relative to native serpentine grassland competitors. *Plant Ecology* 213(6): 1015-1026.

Verlinden M, Nijs I. 2010. Alien plant species favoured over congeneric natives under experimental climate warming in temperate Belgian climate. *Biological Invasions* 12(8): 2777-2787.

Verlinden M, Van Kerkhove A, Nijs I. 2013. Effects of experimental climate warming and associated soil drought on the competition between three highly invasive West European alien plant species and native counterparts. *Plant Ecology* 214(2): 243-254.

Vourlitis GL, Kroon JL. 2013. Growth and resource use of the invasive grass, pampasgrass (*Cortaderia selloana*), in response to nitrogen and water availability. *Weed Science* 61(1): 117-125.

Wang JP, Dong LJ, Sang WG. 2012. Effects of different nitrogen regimes on competition between Ambrosia artemisiifolia, an invasive species, and two native species, Artemisia annua and Artemisia mongolica. *Biodiversity Science* 20(1): 3-11.

Wang ML, Feng YL. 2005. Effects of soil nitrogen levels on morphology, biomass allocationand photosynthesis in *Ageratina adenophora* and *Chromoleana odorata*. *Chinese Jouranl of Plant Ecology* 29(5): 697-705.

Wang RL, Han M, Liang XT, Song YY, Su YJ, Zeng RS. 2011. Responses of biomass allocation

patterns and allelopathic potential of *Bidens pilosa* L. to elevated temperature. *Ecology and Environmental Science* 20(6): 1026-1030.

Wang RL, Zhong QH, Xu WB, Song YY, Su YJ, Zeng RS. 2012. Responses of invasive weed *Mikania micrantha* to elevated air temperature. *Chinese Journal of Ecology* 31(7): 1659-1664.

Wang YT, Li CM, Li SS. 2013. Chilling sensitivities of three closely related plants with different invasiveness in South China. *Acta Ecologica Sinica* 33(18): 5509-5515.

Werner C, Zumkier U, Beyschlag W, Máguas C. 2010. High competitiveness of a resource demanding invasive acacia under low resource supply. *Plant Ecology* 206(1): 83-96.

Williams AL, Wills KE, Janes JK, Vander Schoor JK, Newton PC, Hovenden MJ. 2007. Warming and free-air CO_2 enrichment alter demographics in four co-occurring grassland species. *New Phytologist* 176(2): 365-374.

Williams D, Black R. 1994. Drought response of a native and introduced Hawaiian grass. *Oecologia* 97(4): 512-519.

Xie L. 2009. Effects of warming soil temperature on root, stem and leaf of an invasive species *Alternanthera philoxeroides*. Master thesis, Central China Normal University.

Xu B, Li F, Shan L, Ma Y, Ichizen N, Huang J. 2006. Gas exchange, biomass partition, and water relationships of three grass seedlings under water stress. *Weed Biology and Management* 6(2): 79-88.

Yang JY, Wang YH, Wen GS, Yi LT. 2013. Effects of AM fungi and simulated nitrogen deposition on the growth and biomass accumulation of *Solidago canadensis* seedlings. *Chinese Journal of Ecology* 32(11): 2953-2958.

You W, Yu D, Xie D, Yu L. 2013. Overwintering survival and regrowth of the invasive plant *Eichhornia crassipes* are enhanced by experimental warming in winter. *Aquatic Biology* 19(1): 45-53.

Zhang C, Liu W, Xu Z, Cao H, Ye W. 2006. Responses of vegetative growth and photosynthesis to temperature in the invasive species *Alternanthera philoxeroides* and its indigenous congener *A. sessilis*. *Journal of Tropical and Subtropical Botany* 4(4): 333-339.

Zhang R, Gallagher R, Shea K. 2012. Maternal warming affects early life stages of an invasive thistle. *Plant Biology* 14(5): 783-788.

Zhang WL, Xiao YA, Yan XH, Zhang SS, Huang HQ, Liu BB. 2013. Effects of simulated warming on the growth and reproduction investment of invasive plant *Plantago virginica*. *Chinese Journal of Ecology* 32(11): 2959-2965.

Ziska LH. 2002. Influence of rising atmospheric CO_2 since 1900 on early growth and photosynthetic response of a noxious invasive weed, Canada thistle (Cirsium arvense). *Functional Plant Biology* 29(12): 1387-1392.

Ziska LH. 2003a. Evaluation of the growth response of six invasive species to past, present and future atmospheric carbon dioxide. *Journal of Experimental Botany* 54(381): 395-404.

Ziska LH. 2003b. The impact of nitrogen supply on the potential response of a noxious, invasive weed, Canada thistle (*Cirsium arvense*) to recent increases in atmospheric carbon dioxide. Physiologia Plantarum 119(1): 105-112.

Method S2 Publication bias

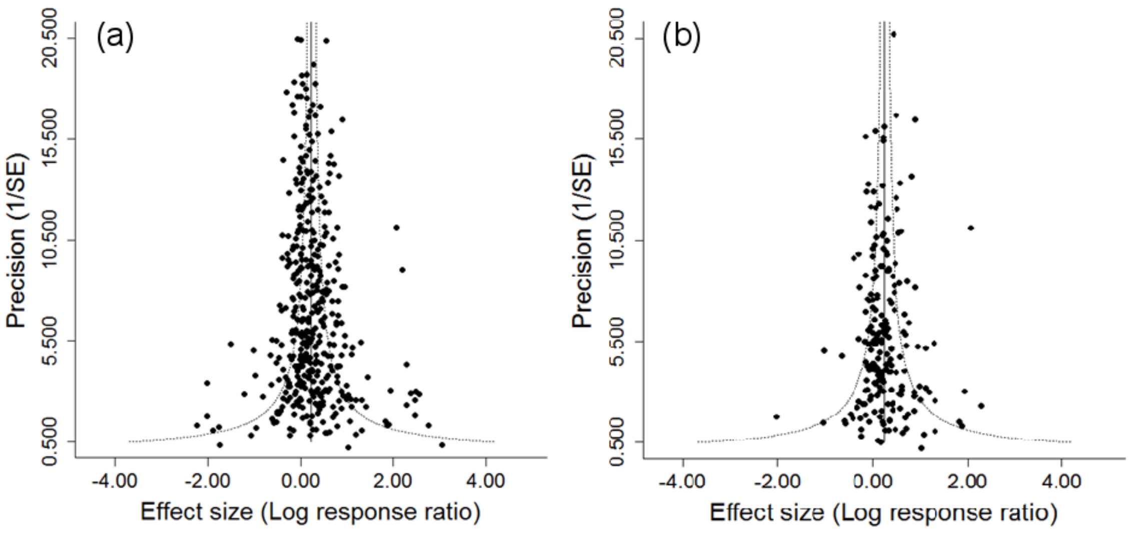

Funnel plot showing the relationship between effect size (ln *R*) and inverse of the standard error. (a) Funnel plot for Data-I. (b) Funnel plot for Data-II.

Method S3 Phylogenetic tree used in this study and list of published studies employed for resolving polytomies within the initial base tree.

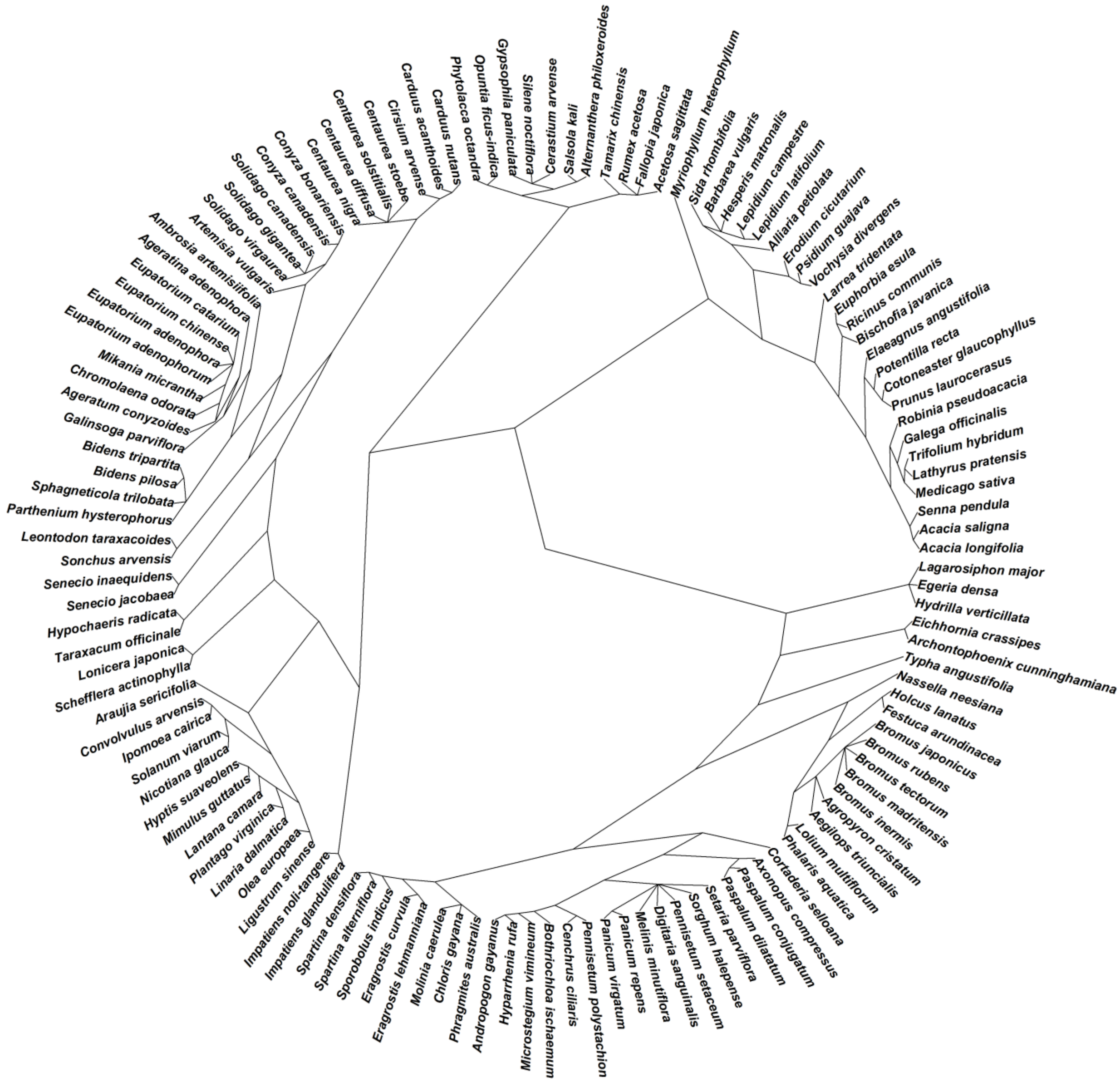

We included phylogenetic relatedness among the study species in models as a random factor by including the variance-covariance matrix of species relatedness. First, a super tree was constructed using the online program, Phylomatic42. Next, we solved polytomies within the base tree using the published molecular phylogenies below. Subsequently, the phylogenetic tree was

transformed to an ultrametric tree using the compute.brlen function in the package ape v 3.2. Finally, we calculated a variance-covariance matrix from the ultrametric tree using the vcv function in the package ape v 3.2, which represents phylogenetic relatedness among species.

Bremer K.R., Friis E. & Bremer B. (2004) Molecular phylogenetic dating of Asterid flowering plants shows early cretaceous diversification. *Systematic Biology*, **53**, 496-505.

Chemisquy M.A., Giussani L.M., Scataglini M.A., Kellogg E.A. & Morrone O. (2010) Phylogenetic studies favour the unification of Pennisetum, Cenchrus and Odontelytrum (Poaceae): a combined nuclear, plastid and morphological analysis, and nomenclatural combinations in Cenchrus. *Annals of Botany*, **106**, 107-130.

Chen C.H., Veldkamp J.F., Kuoh C.S., Tsai C.C. & Chiang Y.C. (2009) Segregation of *Leptatherum* from *Microstegium* (*Andropogoneae*, Poaceae) confirmed by internal transcribed spacer DNA sequences. *Blumea - Biodiversity, Evolution and Biogeography of Plants*, **54**, 175-180.

Dizkirici A., Zeki K., Evren C. & DOĞAN M. (2010) Phylogenetic relationships of *Elymus* L. and related genera (Poaceae) based on the nuclear ribosomal internal transcribed spacer sequences. *Turkish Journal Of Botany*, **34**, 467-478.

Duvall M.R., Davis J.I., Clark L.G., Noll J.D., Goldman D.H. & Sánchez-Ken J.G. (2007) Phylogeny of the grasses (Poaceae) revisited. *Aliso: A Journal of Systematic and Evolutionary Botany*, **23**, 237-247.

Duvall M.R., Saar D.E., Grayburn W.S. & Holbrook G.P. (2003) Complex transitions between C3 and C4 photosynthesis during the evolution of Paniceae: a phylogenetic case study emphasizing the position of *Steinchisma hians* (Poaceae), a C_3-C_4 Intermediate. *International Journal of Plant Sciences*, **164**, 949-958.

Escobar J.S., Scornavacca C., Cenci A., Guilhaumon C., Santoni S., Douzery E.J., Ranwez V., Glémin S. & David J. (2011) Multigenic phylogeny and analysis of tree incongruences in *Triticeae* (Poaceae). *BMC Evolutionary Biology*, **11**, 181.

Goertzen L.R., Cannone J.J., Gutell R.R. & Jansen R.K. (2003) ITS secondary structure derived from comparative analysis: implications for sequence alignment and phylogeny of the Asteraceae. *Molecular Phylogenetics and Evolution*, **29**, 216-234.

Ito M., Yahara T., King R.M., Watanabe K., Oshita S., Yokoyama J. & Crawford D.J. (2000) Molecular phylogeny of Eupatorieae (Asteraceae) estimated from cpDNA RFLP and its implication for the polyploid origin hypothesis of the tribe. *Journal of Plant Research*, **113**, 91-96.

Kumar S., Hahn F.M., McMahan C.M., Cornish K. & Whalen M.C. (2009) Comparative analysis of the complete sequence of the plastid genome of Parthenium argentatum and identification of DNA barcodes to differentiate Parthenium species and lines. *BMC Plant Biol*, **9**, 131.

Liu Q., Peterson P.M., Columbus J.T., Zhao N., Hao G. & Zhang D. (2007) Inflorescence diversification in the "finger millet clade" (Chloridoideae, Poaceae): a comparison of molecular phylogeny and developmental morphology. *American Journal of Botany*, **94**, 1230-1247.

Mason-Gamer R.J. (2013) Phylogeny of a genomically diverse group of elymus (poaceae) allopolyploids reveals multiple levels of reticulation. *PLoS One*, **8**, e78449.

Mathews S., Spangler R.E., Mason-Gamer R.J. & Kellogg E.A. (2002) Phylogeny of Andropogoneae inferred from phytochrome B, GBSSI, and ndhF. *International Journal of Plant Sciences*, **163**, 441-450.

Mathews S., Tsai R.C. & Kellogg E.A. (2000) Phylogenetic structure in the grass family (Poaceae): evidence from the nuclear gene phytochrome B. *American Journal of Botany*, **87**, 96-107.

Morrone O., Aagesen L., Scataglini M.A., Salariato D.L., Denham S.S., Chemisquy M.A., Sede S.M., Giussani L.M., Kellogg E.A. & Zuloaga F.O. (2012) Phylogeny of the Paniceae (Poaceae: Panicoideae): integrating plastid DNA sequences and morphology into a new classification. *Cladistics*, **28**, 333-356.

Nie X., Lv S., Zhang Y., Du X., Wang L., Biradar S.S., Tan X., Wan F. & Weining S. (2012) Complete chloroplast genome sequence of a major invasive species, crofton weed (*Ageratina adenophora*). *PLoS One*, **7**, e36869.

Panero J.L., Jansen R.K. & Clevinger J.A. (1999) Phylogenetic relationships of subtribe Ecliptinae (Asteraceae: Heliantheae) based on chloroplast DNA restriction site data. *American Journal of Botany*, **86**, 413-427.

Petersen G., Seberg O., Yde M. & Berthelsen K. (2006) Phylogenetic relationships of Triticum and Aegilops and evidence for the origin of the A, B, and D genomes of common wheat (*Triticum aestivum*). *Molecular Phylogenetics and Evolution*, **39**, 70-82.

Quintanar A., Castroviejo S. & Catalán P. (2007) Phylogeny of the tribe aveneae (Pooideae, Poaceae) inferred from plastid trnT-F and nuclear ITS sequences. *American Journal of Botany*, **94**, 1554-1569.

Rauscher J.T. (2002) Molecular phylogenetics of the *Espeletia complex* (Asteraceae): evidence from nrDNA ITS sequences on the closest relatives of an Andean adaptive radiation. *American Journal of Botany*, **89**, 1074-1084.

Skendzic E.M., Columbus J.T. & Cerros-Tlatilpa R. (2007) Phylogenetics of Andropogoneae (Poaceae: Panicoideae) based on nuclear ribosomal internal transcribed spacer and chloroplast trnL–F sequences. *Aliso: A Journal of Systematic and Evolutionary Botany*, **23**, 530-544.

Souto D.F., Catalano S., Tosto D., Bernasconi P., Sala A., Wagner M. & Corach D. (2006) Phylogenetic relationships of *Deschampsia antarctica* (Poaceae): Insights from nuclear ribosomal ITS. *Plant Systematics and Evolution*, **261**, 1-9.

van Dijk P.J. (2003) Ecological and evolutionary opportunities of apomixis: insights from Taraxacum and Chondrilla. *Philos Trans R Soc Lond B Biol Sci*, **358**, 1113-1121.

Xu C.M., Qu C.Y., Yu W.G., Zhang X.J. & Li F.Z. (2009) Phylogenetic origin of Beckmannia (Poaceae) inferred from molecular evidence. *Journal of Systematics and Evolution*, **47**, 305-310.

Figure S1. Performance response (indicated by log response ratio mean effect sizes) of alien plants to increased and decreased precipitation.

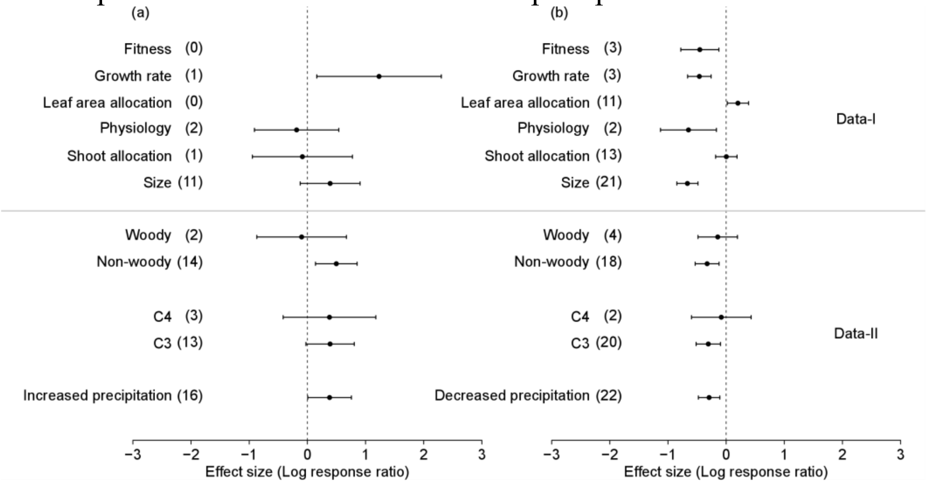

Performance response (indicated by log response ratio of mean effect sizes) of alien plants to increased (a) and decreased precipitation (b). Error bars represent 95% confidence intervals around the mean effect size estimates derived from the phylogenetically informed meta-analytic model. For drought studies, we calculated the effect size by dividing values obtained with drought treatment by those obtained under ambient conditions, whereas for elevated precipitation studies, the effect size was calculated by dividing values obtained with elevated precipitation treatment by those obtained under ambient conditions.
